# Knowledge translation and exercise for degenerative meniscal pathology and early osteoarthritis  (KNEE-DEeP): Protocol for a single arm feasibility study

**DOI:** 10.12688/hrbopenres.14049.1

**Published:** 2025-01-24

**Authors:** Helen O'Leary, Clodagh Toomey, Liam G Ryan, Katie Robinson, Liam Glynn, Helen P French, Karen McCreesh

**Affiliations:** 1 School of Allied Health, Ageing Research Centre, Health Research Institute, University of Limerick, Limerick, County Limerick, V94 T9PX, Ireland; 2 Physiotherapy Department, University Hospital Kerry, Tralee, County Kerry, Ireland; 3 School of Medicine, University of Limerick, Limerick, County Limerick, V94 T9PX, Ireland; 4 School of Physiotherapy, Royal College of Surgeons in Ireland, York Street, Dublin, D02 YN77, Ireland

**Keywords:** knee osteoarthritis, degenerative meniscal tear, feasibility study, exercise therapy, primary care

## Abstract

**Background:**

The Knowledge Translation and Exercise for Degenerative Meniscal Pathology and Early Knee Osteoarthritis (KNEE-DEeP) intervention was designed to promote greater uptake of evidence-based non-surgical treatments for knee pain attributed to degenerative meniscal pathology and early knee osteoarthritis (OA) in primary care, by tackling barriers at a service, clinician and patient level. Evidence indicates that patients frequently do not access first-line treatments, namely exercise and patient education, prior to specialist referral. The KNEE-DEeP intervention supports general practitioners (GPs) and physiotherapists to enhance their skills and confidence in managing patients with this type of knee pain through professional development workshops. In turn, patients will receive an ‘enhanced consultation’ from their GP and be referred to an early ‘best practice’ physiotherapy session. Physiotherapists will work with patients to develop a collaborative action plan focussing on self-management and exercise.

**Methods:**

This protocol outlines a single arm non-randomised feasibility study with a mixed method process evaluation. The study intends to recruit 15 GPs, five physiotherapists and 36 patients from general practices in the South-West of Ireland. Eligible patients, will be aged between 35 years and 69 years inclusive, and attend their GP with an episode of non-traumatic knee pain attributed to a degenerative meniscal tear (DMT) or early OA. Physiotherapists and GPs will be trained in intervention delivery. Within two weeks of receiving an ’enhanced consultation‘ from their participating GP, patients will attend the one-hour ‘best practice’ physiotherapy session. Patient data will be collected via online questionnaires at baseline, 12 weeks and 6 months. Qualitative interviews to assess the feasibility and acceptability of the intervention will be conducted with a purposive sample of GPs, physiotherapists and their enrolled patients.

**Ethics and Dissemination:**

Approved by Clinical Research Ethics Committee of the Cork Teaching Hospitals. Results will be presented in peer-reviewed journals and at international conferences.

**Registration:**

clinicaltrials.gov ( NCT06576557)

## Introduction

The burden of knee pain on healthcare resources and society is increasing ^1^. Knee pain is the most common musculoskeletal presentation seen by UK general practitioners (GPs), after lower back pain ^2^. In the secondary setting in Ireland, knee pain, attributed to knee osteoarthritis (OA) and meniscal pathology are among the most common presentations seen in orthopaedic clinics ^3^. Meniscal tears in adults who are middle-aged and are associated with a range of degenerative joint changes from mild chondral changes to established knee osteoarthritis ^4^. Studies indicate that the presence of a degenerative meniscal tear (DMT) is correlated with the initiation and progression of OA ^4, 5^ and creating a clear line of distinction between these two entities is difficult. Findings of a DMT on magnetic resonance imaging (MRI) of the knee are also common in the general population and increase with age ^6^. Furthermore, there are no clinical features proven to distinguish a DMT from OA. Symptoms traditionally used to diagnose a DMT clinically (localised pain, mechanical symptoms and joint line tenderness) have been proven to be non-specific ^7, 8^ and meniscal tests have limited diagnostic accuracy ^9^.

The reconceptualization of a DMT as part of the continuum of the OA process helps us appreciate why arthroscopic partial meniscectomy (APM) to remove damaged meniscal tissue is not superior to a sham procedure for alleviating symptoms ^10, 11^. Exercise therapy demonstrates clinically important improvements in pain and function in several RCTs that are comparable in effect size to improvements with surgical treatment for a DMT, for both short and long-term follow-up ^12^. Meanwhile some studies suggest APM may even be harmful to overall knee joint health in the longer term ^13, 14^.

Despite recommendations against meniscectomy in favour of conservative treatments like exercise ^15^, APM remains a common orthopaedic procedure worldwide ^16, 17^. In Ireland, patients with knee pain attributed to DMT are frequently referred to orthopaedic clinics for a surgical opinion ^3^, suggesting a sub-optimal use of non-surgical management. Surveys of knee pain attributed to OA show underutilisation of exercise and core treatments before seeking secondary care ^18– 20^. People with knee OA also continue to receive diagnostic interventions such as MRI and therapeutic interventions such as arthroscopy that are contrary to clinical practice guidelines ^21^. The inappropriate use of MRI, has been observed for degenerative knee pain in the Irish healthcare setting ^19, 22^, and its overuse has been implicated in greater healthcare utilisation without yielding better clinical outcomes ^23, 24^. Our qualitative research supports these observed trends, whereby patients with a DMT or early degenerative knee pain, interviewed while awaiting an orthopaedic consultation, reported minimal engagement with exercise therapy, tended to focus on structural pathology reported on MRI and had an expectation of a ‘surgical fix’ to resolve their knee pain ^25^.

Implementing current clinical practice recommendations ^15, 26, 27^ in routine practice is challenging, requiring recognition of barriers at the patient, clinician, and health system levels, as well as local contextual factors ^28^. Our baseline qualitative work explored barriers to delivering evidence-based care for people with early degenerative knee pain or DMT in Ireland with patients, physiotherapists, and GPs ^25, 29, 30^. Patient barriers include unhelpful beliefs about exercise and an understanding that surgery was necessary for long-term knee health. For GPs, gaps in knowledge and confidence, and competing demands during a consultation were some of the reported challenges ^25, 30^. Physiotherapists report barriers like unrealistic patient expectations for recovery time and early patient disengagement from physiotherapy to seek a surgical opinion ^29^. At the health service level, GPs and physiotherapists identified limited access to physiotherapy within the Irish public health service as a significant barrier to patients receiving the recommended exercise therapy for DMT ^30^.

In Ireland, healthcare is a mixed public-private system, with the Health Services Executive (HSE) delivering publicly funded care ^31^. Private sector involvement is substantial, with 44% of citizens having private health insurance ^32^. About 36% of the population is entitled to publicly funded primary care services, but long waits hinder access to timely physiotherapy and community-based services ^31^. As of March 2023, nearly 68,000 patients were waiting for primary care physiotherapy, with 20% waiting over a year ^33^. Patients without entitlement to public physiotherapy pay out-of-pocket, partially reimbursed by private health insurance. Irish GPs expressed a need to "do something" for patients with knee pain facing long physiotherapy waits, triggering earlier referral to secondary care ^30^. This premature escalation places additional demands on public hospital services, where waiting lists for adult orthopaedic service are the longest in Ireland ^34^.

With an appreciation of the barriers to evidence-based care for a DMT and early knee OA, the next stage was to develop a multi-component strategy to address these barriers, using the best available evidence and appropriate theory from the Medical Research Council (MRC) ^35^. The Behavioural Change Wheel (BCW) eight-step process, as described by Michie and colleagues ^36^ guided the design process. The Theoretical Domains Framework (TDF) was used to identify relevant determinants of change and develop an in-depth understanding of each target behaviour ^37^. Whilst existing evidence specifically on the implementation of DMT clinical recommendations is lacking, we drew on the closely related OA literature to help inform our intervention development process. According to a recent scoping review, primary care initiatives to support the delivery of evidence-based care for OA are typically GP-led, and also entail referral to other allied health services such as physiotherapy ^38^. Some previous implementation strategies designed to improve the delivery of evidence-based care for knee OA have focused on enhancing the initial consultation between the patients and clinician in the primary care setting ^39– 41^. Education and training for GPs delivering this initial consultation is an important component of these care models ^38^. These model OA consultations have demonstrated improved uptake of evidence-based care ^39, 42^, and in some instances reduced healthcare usage ^43^ but not necessarily improved clinical outcomes compared to usual care ^38^. Nonetheless a key focus of our feasibility study is to promote use of low cost intervention such as education and exercise, thereby reducing unnecessary secondary care referral. Given the emphasis placed by GPs on timely access to physiotherapy in managing this condition, early access to a single ‘best practice’ session of physiotherapy will be a key component of the intervention, to address this service-level barrier. While a single session will not fulfil all patients’ needs, early physiotherapy assessment and education shows promise for reducing downstream health care utilisation ^44^, and was found as effective as multiple sessions in some musculoskeletal conditions ^45, 46^. For interventions to be successfully implemented, stakeholder involvement is key to understanding the context locally and ensuring proposed interventions are acceptable, engaging and feasible ^47^, and we consulted widely with people with knee pain and clinicians (GPs, physiotherapists and orthopaedic consultants). Further details on the key features and development of the study intervention have been presented elsewhere ^48^ and a full manuscript is in preparation.

Our final intervention, called the KNEE-DEeP (Knowledge Translation and Exercise for Degenerative Meniscal Pathology and Early Knee Osteoarthritis) intervention, will target both GPs and physiotherapists, and their patients with knee pain attributed to early OA or a DMT *.* The patient targeted intervention will be an ‘enhanced consultation’ with their participating GP and referral for a single early ’best practice’ physiotherapy session. Participating GPs and physiotherapists will receive training and educational resources to facilitate these clinical consultations. Given the evidence that knee OA and DMTs frequently co-exist, and are essentially part of the same disease process, this study opted to include patients with DMTs and early OA, while excluding people with established knee OA, based on clinical criteria ^49^. Several RCTs investigating surgery for DMT have also included people with some evidence of knee OA ^50– 52^, while excluding people with advanced knee OA.

The next step is to understand if this complex intervention is feasible to deliver in a ‘real-world’ setting. The community setting and general practice has many competing demands. It is essential to understand how the intervention will interact within the context of general practice and if implementing early access to a single ‘best practice’ session is feasible within a publicly funded physiotherapy service ^35^. Uncertainties about the acceptability of the intervention to HCPs and patients also need to be resolved. To a lesser extent we also want to investigate aspects of feasibility related to recruitment and retention of both HCPs and patients. To investigate these key components we opted to conduct a single-arm feasibility study and did not randomise or include a control group.

### Aims

The aim of this study is to test the feasibility of the KNEE-DEeP intervention, a complex health intervention targeted at both HCPs and patients, and designed to promote greater uptake of evidence based care for DMT and early OA.

Specifically, the objectives are to:

1. Determine the acceptability of the intervention content and delivery to HCPs and patients
2. Determine if the HCP intervention (training workshops) is feasible to deliver to GPs and physiotherapists
3. Determine if the components of the patient intervention (‘enhanced GP consultation’ and ‘best practice’ physiotherapy session) are feasible to deliver within general practice and a publicly funded physiotherapy service.
4. Assess fidelity related to intervention delivery and intervention receipt, from the perspectives of HCPs and patients.
5. Determine if data collection procedures and outcome measures are feasible and acceptable to HCPs and patients
6. Determine compliance of GPs with study processes and procedures around recruitment and referral of patients
7. Determine feasibility of HCP and patient recruitment and retention procedures.
8. Investigate the magnitude of change and variability in the clinical outcome measures.

## Methods

### Study design

The KNEE-DEeP study is a non-randomised feasibility study that includes an embedded mixed methods process evaluation. The KNEE-DEeP knowledge translation intervention will be targeted at recruited GPs ad physiotherapists, who in turn will deliver the patient intervention to people recruited with knee pain. Quantitative and qualitative data will be collected from GPs, physiotherapists and patients as part of the process evaluation. Assessments will be performed at baseline, 12 weeks (primary endpoint) and 6 months. Recruitment is anticipated to last 6 months, with each patient participant taking part in the study for 6 months and the last follow-up due 12 months after the start of recruitment.

**Figure 1.**
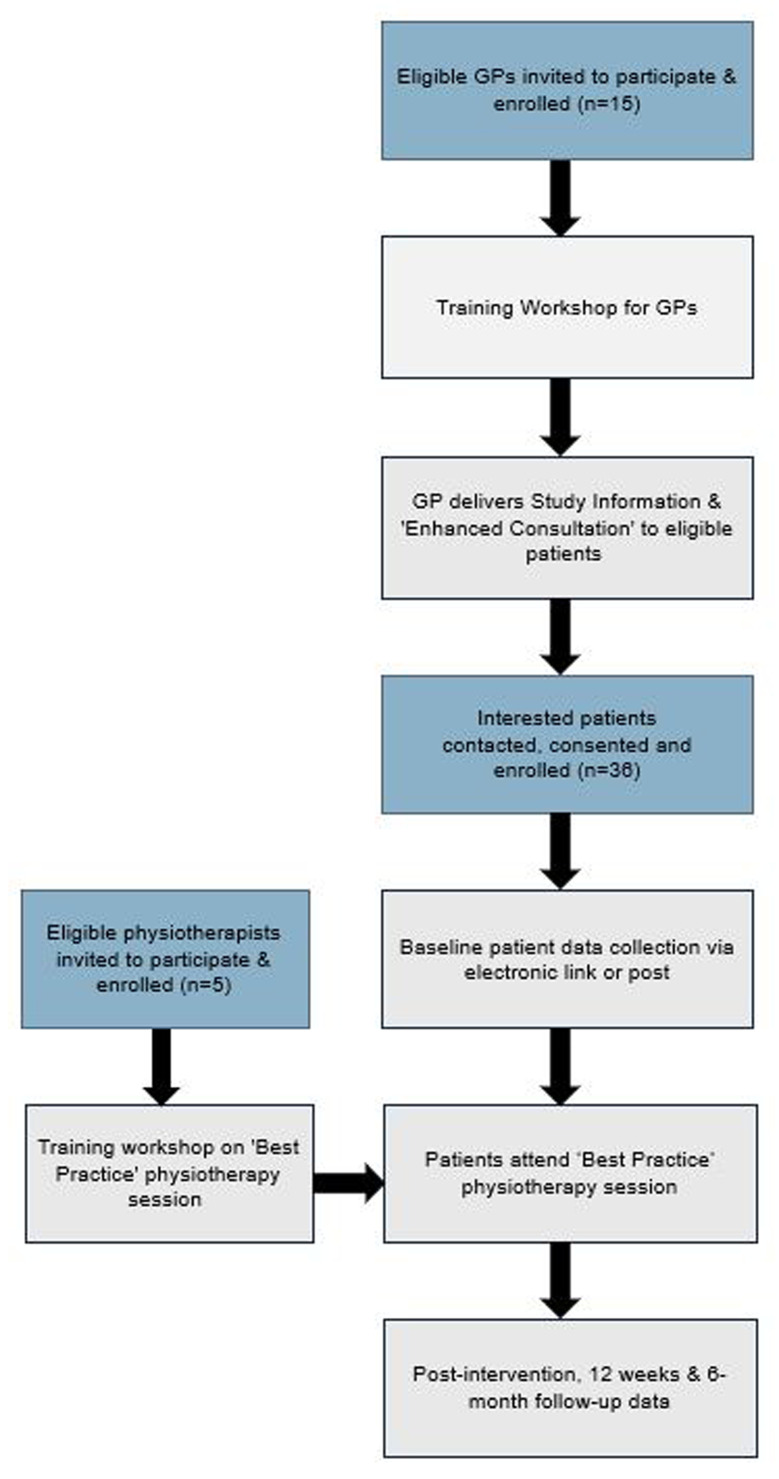
Study Flow Diagram.

 This study will be conducted and reported in line with the Standard Protocol Items for Intervention Trials (SPIRIT) guidelines and the Consolidated Standards of Reporting Trials ^53, 54^. Completed checklists are included in the extended data (File A and B, https://osf.io/fwy36/). The study is registered with ClinicalTrials.gov (Identifier: NCT06576557). Further evaluation work may be necessary to test the feasibility of key study design and processes for a future full-scale effectiveness trial. Figure 1 illustrates the flow of participants through the study and Table 1 outlines the schedule of enrolment, intervention and assessments.

**Table 1.** Schedule of study enrolment, interventions and assessment.

**Timepoint**	-T _3_	-T _2_	-T _1_	T _0_	0–2 week	2–12 weeks	12 week	6 months
Enrolment								
GP enrolment	x							
Physio enrolment	x							
Pt enrolment				x				
Intervention								
GP training		x						
Physio training		x						
GP ‘Enhanced Consultation’			x					
‘Best practice’ Physio Session					x			
Assessment								
*Quantitative Data Collection*								
GP & Physio descriptive data		x						
Pt baseline descriptive data				x				
Pt clinical & feasibility outcomes				x			x	x
GP & Physio follow-up questionnaire								x
*Qualitative Data collection*								
Pt Interviews							x	x
GP & Physio Interviews								x

GP, general practitioner; Physio, physiotherapist; Pt, patient; T _0_, baseline.

This study is located within the feasibility and piloting stage of the MRC’s framework that provides guidance on the design and development of complex interventions ^35^. A single arm feasibility study was chosen rather than a comparative design because the main focus was to resolve uncertainties about the acceptability and feasibility of the intervention itself, and its mode of delivery. A non-randomised design provided an opportunity to test the intervention with a greater number of HCP and patient participants. According to the MRC’s guidance on the development and evaluation of complex healthcare interventions, a feasibility study need not be a scale model of a future planned evaluation but instead is an opportunity to examine the key uncertainties that have been identified during development ^35, 55^.

### Setting

This multi-site study is set in general practices in Kerry and North Cork and in the Physiotherapy Department of University Hospital Kerry (UHK), a publicly funded hospital also in the South-West of Ireland.

### Public and Patient Involvement

A patient and public involvement (PPI) group was established and have been involved in all aspects of the intervention design phase and planning for this feasibility study. This group comprises of six people who had previously engaged with both primary and secondary care health services for treatment of knee pain attributed to early degenerative changes or knee OA. Two members have previously undergone arthroscopic knee surgery for DMT. This PPI group have met four times and their perspective was integrated into the intervention and study design process. They will continue to be consulted over the course of the feasibility study.

### Ethics and informed consent

Ethical approval for this study was granted by Clinical Research Ethics Committee of the Cork Teaching Hospitals on the 5 ^th^ December 2023 ( *Reference Number: ECM 4 (h) 24/10/2023 & ECM 3 (d) 05/12/2023)*. This committee has remit to provide approval for studies conducted within the HSE Community Health Organisation for Cork/Kerry. This study involves human participants and will adhere to the Declaration of Helsinki ^56^. Prior to study recruitment all participants will provide written informed consent, in line with the Data Protection Act 2018 ^57^. Eligible participants will be invited to ask questions about the study and a time period of several days will elapse between receiving information about the study and making their final decision about whether to participate.

### Recruitment and participants

***General practitioners.*** Fifteen GPs will be recruited by contacting local general medical practices known to the research team with study information. Networks such as the Health Research Board (HRB) Primary Care Clinical Trials Network Ireland will also be used to circulate information about the study to practices. Eligible GPs will be practicing in Kerry or North Cork and within traveling distance (< 50 km) of the hospital providing the physiotherapy intervention. It is estimated there are approximately 30 eligible practices. Practices where two or more GPs are willing to participate will be preferentially recruited and recruitment will aim for a geographical spread. At least 15 interested GPs will be recruited. The principal investigator (PI) (HOL) will answer any questions by telephone or on a subsequent practice visit, prior to obtaining consent from each GP (Extended data, File C, https://osf.io/fwy36/) ^58^ individually and enrolling them in the study.

***Physiotherapists.*** Five physiotherapists will be recruited from the Physiotherapy Outpatient Department of a local publicly funded hospital (UHK) where participating patients will receive their physiotherapy intervention. Given the small size of the study, for pragmatic reasons in order to train sufficient physiotherapy staff, we opted to have all patients receive their intervention in one central and accessible location. The physiotherapy manager will facilitate recruitment of physiotherapists (n=5) by providing study information to all eligible physiotherapists in UHK (n=8). Eligible physiotherapists will be involved in the delivery of outpatient musculoskeletal physiotherapy services and available to participate in training to deliver the ‘best practice’ physiotherapy intervention. Having considered the study information, interested physiotherapists will make contact with the PI, who will answer any questions pertaining to the study, prior to them signing a consent form (Extended Data, File D, https://osf.io/fwy36/) ^58^ and enrolling in the study.

***Patients with knee pain.*** Study information will be displayed in the patient waiting areas of the participating general practices. During their consultation, participating GPs will inform eligible patients about the study and provide them with written study information (Extended Data, File E, https://osf.io/fwy36/) ^58^. Patients will also receive verbal and written educational information on their knee pain prior to enrolling in the study, representing an enhanced version of usual care. As part of usual care for people attending their GP with knee pain, patients are routinely assessed and educated about their condition ^30^, therefore this intervention does not represent any risk or burden to patients who ultimately do not enrol in the study and decline to participate in data collection. GPs will also explain that study participation involves attending a ‘best practice’ session of physiotherapy in UHK and this not affect the patient’s access to a routine physiotherapy appointment (typically 6 to 12 months wait for a public appointment), should patients also wish to avail of this. Having discussed the study, GPs will seek written consent from interested patients to forward their contact details to the research team. The PI will contact prospective participants by telephone to answer any questions, confirm their eligibility and that they have read the written study information. Consenting patients will be enrolled in the study, following receipt of a signed consent form either written or electronically. An emailed link to the baseline questionnaires will follow. Participants will also indicate if they are amenable to being contacted for a post-intervention qualitative interview. They will sign a subsequent consent form before this interview is conducted.

If a potential participant ultimately declines to participate, having discussed the study with the researcher, they will still have the opportunity to avail of the ‘best practice’ physiotherapy intervention, but no study data will be collected. 

Patients will be eligible for this study if they are:

- Attending their GP with an episode of non-traumatic knee pain.
- Have knee pain attributed to a diagnosis of DMT and early OA, made via a GP clinical assessment and based on features agreed by expert consensus ^59, 60^.
- Aged between 35 and 69 years inclusive. Inclusion of people aged 35 years or older is based on expert consensus, clinical practice guidelines and several RCTs investigating surgery for people with degenerative meniscal lesions also use this age cut-off ^10, 15, 59, 61^.

Criteria for exclusion are: (i) fulfilling the European League Against Rheumatism (EULAR) clinical classification criteria (Proposition No. 5) for knee OA, which reflect more established OA disease ^49^, (ii) moderate or advanced knee OA on x-ray (or Kellgren-Lawrence x-ray score ≥ Grade 3), (iii) recent trauma likely to be associated with considerable tissue damage, (iv) having an acutely swollen or locked knee, or suspicion of ligament injury on physical exam, (v) inflammatory arthritis, (vi) surgery or significant trauma of the index knee within the previous 2 years, (vii) pregnancy, (viii) unable to communicate in English (ix) preference for accessing physiotherapy treatment privately prior to the ‘best practice’ session or do not want to avail of the ‘best practice’ physiotherapy session.

There is a clear association between knee OA and DMTs, and meniscal pathology usually exists in the context of other OA changes ^5, 59, 62^. The EULAR clinical classification criteria will be used by GPs to rule out later stage or more advanced OA during their clinical assessment ^49^. These criteria state that patients aged 40 years or older with movement-related joint pain, knee morning stiffness < 30 minutes, and functional limitations have knee OA, if they also have, on examination, one or more of the following: crepitus, restricted range of motion, and bony enlargement. Should there be uncertainty on clinical exam, a GP-ordered knee x-ray will be necessary for inclusion, to exclude those with moderate or severe osteoarthritis (Kellgren-Lawrence score ≥ Grade 3). Assessment procedures will be a specific component of the training workshop for participating GPs. Patients will have their diagnosis of a DMT or early OA confirmed by the participating physiotherapists, who have expertise in musculoskeletal assessment. They will use a checklist to check and rule out any features of moderate or advanced OA. This research will promote appropriate use of advanced imaging; therefore an MRI will not be required to confirm the diagnosis of a DMT or early OA ^27^.

***Sample size and recruitment targets.*** As this is a feasibility study and the primary aim is not to evaluate clinical effectiveness, a formal sample size calculation has not been carried out ^54, 63^. The purpose of the study is to investigate the feasibility and acceptability of the intervention and examine study procedures around recruitment and retention with a view to refining these, if necessary, for a future definitive trial. The feasibility of the intervention will be explored by recruiting 36 eligible patients, 15 GPs and five physiotherapists.

Based on consultations with local GPs, on average, two new patients present per month with a DMT or early OA, permitting a target of one patient recruited per GP per month. Twelve GPs should recruit 36 patients over 4 months. Allowing for some GPs who will not recruit any patients we set a recruitment target of 15 GPs. General practices are likely to be enrolled on a staggered basis; therefore the patient recruitment phase will be of approximately six months duration.

### Intervention for health care professional and patient participants

**Figure 2.**
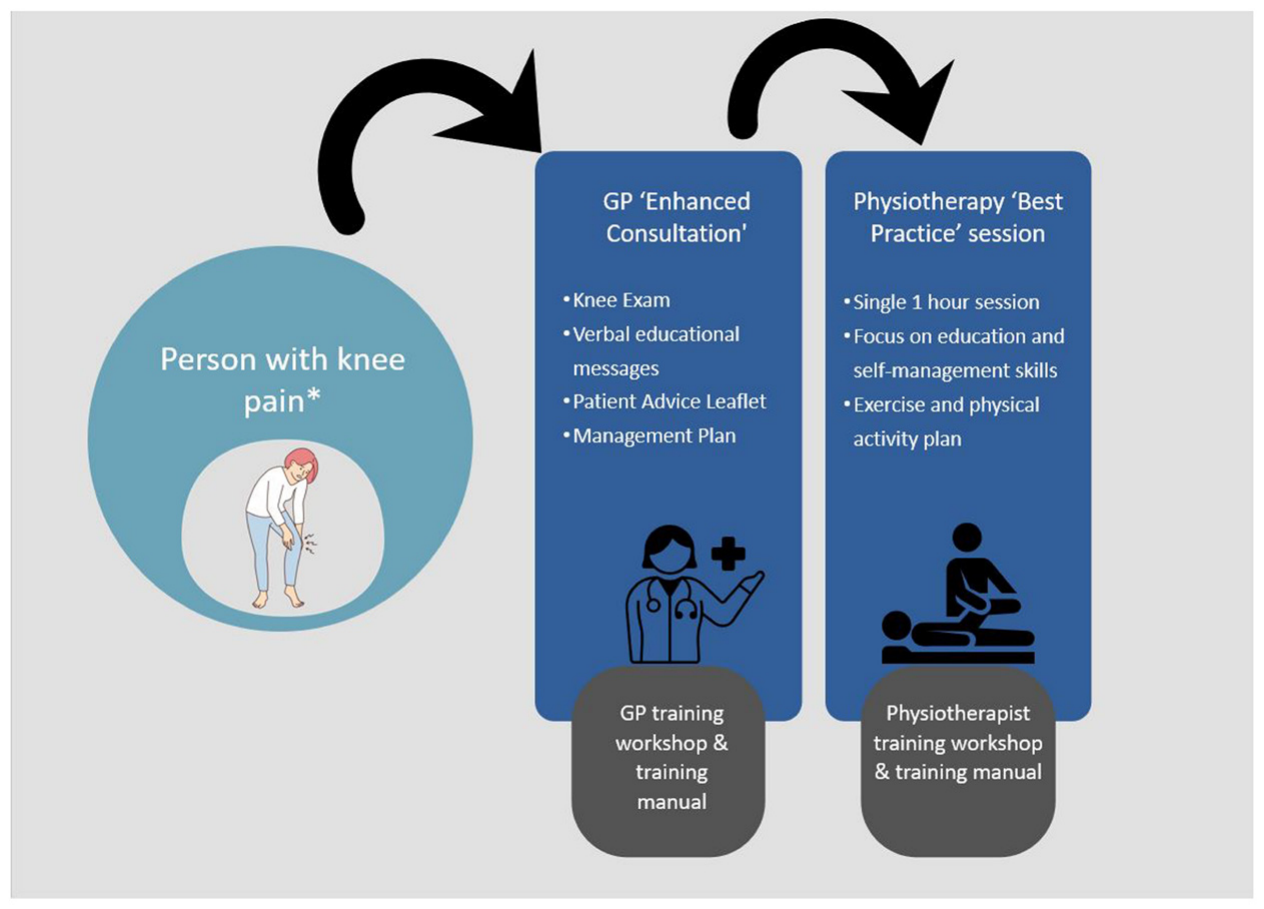
KNEE-DEeP Intervention. * degenerative meniscal pathology or early osteoarthritis

The KNEE-DEeP intervention ( Figure 2) is a complex intervention designed to improve the uptake of evidence-based care for early OA and DMTs, by targeting the behaviours of both HCPs and their patients ^48^. Evidence based care is informed by clinical practice guidelines and focuses on patient education, exercise and promoting self-management ^11, 15, 26^. The KNEE-DEeP intervention will aim to ensure patients receive evidence based management and consistent information at an early point in their care from both the GP and physiotherapist, thereby reducing unnecessary referral to secondary care and inappropriate imaging. GPs and physiotherapists will receive specific training to deliver the patient intervention and enhance their skills and confidence in managing patients with this type of knee pain. Content and design of the HCPs training session will draw on evidence from a range of chronic musculoskeletal conditions ^64– 69^. The content of the intervention aligning to the Template for Intervention Description and Replication (TIDieR) guidelines is summarised in Table 2 ^70^.

**Table 2.** Overview of KNEE-DEeP intervention delivery described according to the TIDieR guidelines.

	Item
1.	Brief name
	Knowledge Translation and Exercise for Degenerative Meniscal Pathology and Early Knee Osteoarthritis (KNEE-DEeP)
2.	Why
	Knee pain in people with degenerative meniscal pathology and early OA is frequently managed sub-optimally with underuse of evidence-based treatments like exercise and education, and overuse of orthopaedic referrals and advanced imaging. The KNEE-DEeP intervention was designed by integrating behavioural change theory with qualitative findings from clinicians and patients and adapted to the local health context. The intervention is targeted at both patients and health care professionals to improve the uptake of evidence-based care for patients with early knee OA and degenerative meniscal pathology.
3.	What (intervention procedures)
	(ii) GPs attend a 2-hour educational workshop on management of this type of knee pain and receive written educational resources. Physiotherapists attend a 2-hour training session supplemented with a pre-recorded webinar and written educational materials on delivery of the ’best practice’ physiotherapy session. Training content will be based on clinical guidelines for knee OA and degenerative knee disease and the wider musculoskeletal evidence base on self-management and parson centred care. (ii) Patient participants receive an ‘enhanced consultation’ from their GP. This constitutes a physical examination, key educational messages, a Patient Advice Leaflet “Understanding My Knee Pain”, a simple starter exercise programme, a management plan agreed with the GP and referral to the ‘best practice’ physiotherapy session. Patient participants also attend a 1-hour best practice physiotherapy session with a participating physiotherapist. The core elements of the session are (i) a clinical assessment (ii) an exploration of the patient’s beliefs and expectations about their knee pain (iii) tailored patient education on likely prognosis, beliefs about pain, physical activity, weight management and other lifestyle factors (iv) information on self-management skills such as load management, activity modification and monitoring pain response (v) completion of a goal and action plan worksheet (vi) provision of an exercise programme and/or physical activity plan.
3.	What (materials)
	GPs receive a study folder handbook detailing study procedures (e.g. recruitment, intervention delivery, referral to ‘best practice’ physiotherapy session). Educational materials will include copy of PowerPoint slides, communication tips, links to patient educational videos on weight maintenance, load management etc. Physiotherapists receive a training manual detailing components of the ‘best practice’ physiotherapy session, a pre-recorded webinar on addressing patient fears and misconceptions about their knee pain, and educational materials and self-management resources. Participating patients receive a Patient Advice Leaflet, with illustrated home exercises and video links, written exercise programme with progressions and regressions, a goal setting and action plan worksheet, physical activity logbook and additional educational handouts on management of flare-ups etc, as appropriate.
4.	Who provided
	Participating GPs deliver the ‘enhanced consultation’ to potential patient participants, while participating musculoskeletal physiotherapists provide the ‘best practice’ physiotherapy session. A clinical specialist physiotherapist and researcher (HOL) facilitates the training workshops to participating GP and physiotherapists, with support from clinical and academic physiotherapy colleagues.
5.	How
	Training workshops are delivered to GPs in small groups (n=6 to 8), either as in-practice training, or with several GPs practices together. Participating patients receive an ‘enhanced consultation’ during a routine consultation with their GP and the ‘best practice’ session will be one-to-one with the physiotherapist.
6.	Where
	The location for GP training varies, either in a GP practice or a rented meeting room to facilitate GPs from several practices. Physiotherapist training is held in the outpatient clinic of a physiotherapy department in a publicly funded hospital, where the ‘best practice’ physiotherapy intervention is delivered. GPs deliver an ‘enhanced consultation’ in their practice.
7.	When and how much
	Participating GPs and physiotherapists receive their respective 2-hour training workshop on enrollment into the study. Patient participants receive an ‘enhanced consultation’ with their participating GP when they present to their GP practice with knee pain. A single one-hour ‘best practice’ physiotherapy session is delivered within 2 weeks of the GP consultation.
8.	Tailoring
	Participating GPs are advised to individualise all aspects of their advice and education according to patients' presentation. Tailoring of patient education and individualised exercise prescription will be a key aspect of the physiotherapy intervention according to patients' goals and preferences.
9.	Modifications
	Any modifications will be reported.
10.	How well (planned)
	Participating GPs physiotherapists receive prior training in how to deliver the patient intervention. Fidelity checklists to self-assess their delivery are completed by GPs and physiotherapists. Participating patients complete a checklist of components of intervention received during the GP consultation.
11.	How well (actual)
	This will be reported in the primary paper.

OA, osteoarthritis; TIDieR, Template for Intervention Description and Replication.

***GP intervention.*** The main components of the GP intervention are a training workshop to support the delivery of an ‘enhanced consultation’ for this type of knee pain, a Patient Advice Leaflet to support their consultation, and the facility to refer eligible patients to a ‘best practice’ physiotherapy session.

a) **GP training workshops** are focused on training and supporting GPs to deliver an ‘enhanced consultation’ for knee pain. Training will be delivered in-person by the PI and supported by two experienced musculoskeletal physiotherapists. This 2-hour training session will cover; physical examination of the knee, clinical features of degenerative meniscal pathology and early OA, screening for serious pathology, appropriate use of imaging ^27^, information on evidence-based treatment options and exercise prescription. Tips on communicating effectively with patients and providing a diagnosis will be discussed. GPs will be introduced to four key educational messages to provide within the patient consultation, and how to use the Patient Advice Leaflet to prescribe some simple exercises. Other components of the ‘enhanced consultation’ including a physical examination and making a treatment plan will be outlined, along with the KNEE-DEeP study procedure. An online training session will be held to facilitate GPs unable to attend in-person. Training content will remain consistent by following a training manual and using PowerPoint slides. Workshops will be accredited by the Irish College of General Practitioners and GPs will receive Continuous Professional Development credits for attending. Study procedures around patient recruitment, along with additional information and resources for GPs to use with patients, will be provided in study folders provided to each individual GP.
b) A **Patient advice leaflet** (Extended Data, File F, https://osf.io/fwy36/) ^58^ written in plain language will aim to support educational messages delivered verbally and provide the GP with a means to prescribe some simple ‘starter’ exercises. This bespoke educational resource designed with patient stakeholders will re-enforce key messages delivered verbally on diagnosis, prognosis and management. Knee exercises organised into three levels of difficultly are described in the leaflet and accompanied by a QR code linking to exercise videos.
c) GPs will have the facility to refer eligible patients to an early **‘best practice’ physiotherapy session**. GPs will complete a bespoke physiotherapy referral form and email to the Physiotherapy Department of UHK. The referral form (Extended Data, File G, https://osf.io/fwy36/) ^58^ will prompt GPs to indicate that they have provided the components of an ‘enhanced consultation’ prior to making the referral. An in-person physiotherapy appointment will follow within two weeks. The components of the GP ‘enhanced consultation’ and the content of the ‘best practice’ physiotherapy session are described below.

***Physiotherapy intervention.*** This intervention for participating physiotherapists consists of training and provision of educational resources to support the delivery of the ‘best practice’ physiotherapy session.

a) A **physiotherapist training workshop** will be delivered by two members of the research team (HOL and KMC). This two-hour session will include review of knee joint assessment, exploring individual patient beliefs and expectations about their knee pain, delivering tailored patient education, exercise prescription, self-management skills and techniques to maximise patient adherence to a management plan. The workshop will focus on training physiotherapist to adopt a collaborative approach during the ‘best practice’ session that focuses on listening to the patient, understanding their individual concerns and worries, delivering information to reduce fear and address misconceptions, before designing a plan focused on their own goals and interests and delivering tailored education and an exercise programme. This session will be accompanied by a training manual to ensure standardisation of training and material delivered. While training content for clinicians is standardised, physiotherapists will be encouraged to individualise the patient sessions. In addition to the training workshop, physiotherapists will listen to a thirty-minute pre-recorded webinar on addressing common patient misconceptions and beliefs about knee pain. The training manual will include written guidance on the core components of the ‘best practice’ session and additional educational resources on knee pain and communication tips.

***Patient intervention.*** The patient intervention consists of an ‘enhanced consultation’ with their GP and a ‘best practice’ physiotherapy session delivered within two weeks of their GP visit. The patient intervention will be guided by clinical guidelines and include education, exercise and where appropriate, weight management advice ^26^. A core focus will be boosting self-management skills and empowering patients with information and resources to manage their symptoms, without the need for inappropriate imaging or surgical consultations. These intervention components will be delivered briefly during the GP consultation through verbal and written information, and addressed more comprehensively during the physiotherapy ‘best practice’ session and using printed and video resources.

a) The ‘ **enhanced consultation’** with the GP consists of four components; (i) a clinical assessment, (ii) verbally delivered educational messages, (iii) a Patient Advice Leaflet and (iv) a documented treatment plan between the GP and patient. The key educational messages relate to providing a diagnosis, setting positive realistic expectations about prognosis, the safety and benefits of exercise for the knee, and tips for long-term knee health including, as appropriate, weight maintenance or weight loss (Extended Data, File H, https://osf.io/fwy36/) ^58^. GPs guidance on the consultation will be to provide reassurance to patients through a thorough clinical examination and providing a broad diagnosis for knee pain that draws on the patient history and potential risk factors, rather than a narrow focus on structural pathology. Encouraging positive patient attitudes about the role of exercise prior to attending the physiotherapy session is another important tenant.
b) The early **‘best practice’ physiotherapy** session is a single face-to-face appointment with a participating physiotherapist in the Physiotherapy Department. This single session is not designed to replace usual physiotherapy care, rather it aims to re-enforce the brief educational messages delivered by the GP and empower the patient through education and self-management skills to take early and positive steps towards their own recovery. The structured one-hour session with the physiotherapist will include the following core elements (i) a clinical assessment (ii) an exploration of individual patient beliefs and expectations about their knee pain and addressing misconceptions (iii) provision of individualised patient education on likely prognosis, beliefs about pain, physical activity, weight management and other lifestyle factors (iv) education on self-management skills such as load management, activity modification and monitoring pain response (v) completion of a goal and action plan worksheet. Patients will collaboratively identify specific personalised goals and targets with the physiotherapist and develop a linked action plan that includes a home exercise programme and /or a physical activity plan. Exercises are selected from a menu (Extended Data, File I, https://osf.io/fwy36/) ^58^ that includes simple starter exercises and more advanced exercises. Organised into three levels of difficulty, these exercises target the hip and knee muscles and will be selected according to individual patients goals and preferences. Guidance will be provided on progressing and regressing individual exercises. Participants will be encouraged to use a physical activity logbook to self-monitor progress and adherence. Depending on their educational needs they may receive additional written resources (e.g managing flare-ups).
  Having availed of the study intervention, it will be at the discretion of the patient and their GP whether to arrange for further physiotherapy, either public or private, depending on the accessibility of their local services. The physiotherapist delivering the ‘best practice’ session can make a recommendation to the GP on the discharge summary. Any further treatment the patient avails of will be recorded by the follow-up patient questionnaires. During the 6-month follow-up period, patients can contact their physiotherapist over the phone for further advice or sign-posting.

**Relevant concomitant care permitted or prohibited during the study**

During the follow-up period patient participants are not precluded from receiving other non-surgical treatments for their knee pain (e.g pain medication, additional physiotherapy). Utilisation of these co-interventions will be captured by the health utilisation questionnaire at 12 weeks and 6 months. Accessing knee surgery (i.e. arthroscopy) during the follow-up period would result in the patient being excluded from the study.

### Outcomes

**Feasibility and implementation outcomes**

Data will be collect on implementation and feasibility outcomes to inform decisions about the need for further evaluation of the KNEE-DEeP intervention and a fully-powered trial. Proctor and colleagues framework on outcomes for implementation research was used to select a series of appropriate feasibility and implementation outcomes ^71^. Data will be collected on intervention feasibility, intervention acceptability, the feasibility and acceptability of study procedures, intervention fidelity, and preliminary efficacy. Quantitative data using self-report questionnaires and qualitative interview data from both HCPs and patients will be integrated to gain a comprehensive understanding of intervention delivery and implementation processes. Incorporating qualitative data collection is recommended given the capacity to this type of data to add depth to the interpretation of such feasibility study evaluations ^72^. Table 1 illustrates the timing of quantitative and qualitative data collection. Table 3 summarises all data collection from patient participants.

**Table 3.** Summary of quantitative data collection from patient participants.

Domain	Measurement Tool	Baseline	12 weeks	24 weeks
*Descriptive data*				
Age, gender, self-report weight and height		X		
Education level		X		
Employment status and occupation		X		
Current pain medication use		X		
History of knee injury, kneeling occupation or previous surgery		X		
Symptom duration				
Other knee symptoms		X		
Other pain sites		X		
Comorbidities		X		
*Primary Clinical Outcome Measures*				
Knee function	KOOS-PS	X	X	X
*Secondary Clinical Outcome Measures*				
Knee pain	KOOS pain subscale	X	X	X
Pain self-efficacy	PSEQ-2	X	X	X
Fear avoidance beliefs	FABQ	X	X	X
Health related quality of life	5Q-5D-5L	X	X	X
Satisfaction with care	Self-rated on 7-point ordinal scale for GP and physiotherapy care		X	
Global rating of change	Self-rated on 7-point ordinal scale		X	X
Number of working days missed	Self-reported			
*Additional Measure*s				
Recovery Expectations	5-point ordinal scale	X		
Adherence to the exercise and physical activity plan	Self-rated on 11-point NRS		X	
Data on healthcare utilisation	Self-reported use of health services and co-interventions		X	X
Change in pain medication use	Self-rated on 7-point Likert scale		X	X
Intervention acceptability	TFA Questionnaire		X	
Harms	Self-report of adverse events		X	X

EQ-5D-5L, EuroQol-5 Dimensions-5 Levels; FABQ, Fear Avoidance Beliefs Questionnaire; KOOS, Knee Injury and Osteoarthritis Outcome Score; KOOS-PS, Knee Injury and Osteoarthritis Outcome Score Physical Function Short Form; NRS, Numerical Rating Scale; PSEQ-2, Pain Self-Efficacy Scale Questionnaire 2 item short form; TFA, Theoretical Framework of Acceptability.

***KNEE-DEeP intervention feasibility.*** Feasibility is the extent to which a new treatment or an innovation can be successfully used or carried out in a given setting ^71^. A feasibility questionnaire containing a previously validated measure to assess intervention feasibility will be completed by participating physiotherapists and GPs ^73^. The Feasibility of Intervention Measure (FIM) is a 4-item scale that measures the extent to which a new intervention can be successfully delivered within a given setting. Items are measured on a 5-point Likert scale (Completely Disagree-Completely Agree) and a mean score is calculated.

Qualitative one-to one interviews with HCPs will explore aspects of feasibility, including if the GP ‘enhanced consultation’ and ‘best practice’ physiotherapy session were practical and feasible for the respective HCPs to deliver. HCPs perspectives on how, why and for whom the interventions did or did not work, and possible barriers to future delivery of the intervention within the broader health care system will also be explored through these interviews. Patients will be interviewed to understand their perceptions of the relevance, usefulness and feasibility of the intervention. Whether participants felt their confidence and ability to manage their knee pain had changed in response to the intervention will also be explored.

***Feasibility and acceptability of KNEE-DEeP study procedures.*** Data collected on feasibility of recruitment will include the number of eligible GP practices and GPs approached, the number of GPs attending the educational workshop who consented to participate, the number of patients screened by the GP who consented to be contacted by the researcher, the number of patients contacted by the researcher who consented to participating, reasons for declining to participate or ineligibility (logged by either participating GP or researcher responsible for enrolling patients) and the number of recruited participants who attended the ‘best practice’ physiotherapy intervention. The retention rate is the proportion (%) of enrolled patients who complete the 12-week (primary endpoint) and 6-month follow-up. Retention rate for GPs will be the proportion who engaged with the study by referring at least one patient to the ‘best practice’ physiotherapy session by the end of the recruitment period. The follow-up response rate is the proportion (%) of returned outcome forms at 12-weeks and 6-month follow-up and the proportion (%) of missing data. Data will be collected on adherence to study procedures around the referral to physiotherapy and completeness of referral forms and other study documentation by GPs and physiotherapists adherence to protocols around these.

Qualitative data from individual GP interviews will enhance quantitative data collection, and explore barriers and facilitators to recruitment. It will also explore the ease of procedures like explaining the study to patients and referring to the physiotherapy session. Patients will be asked about what motivated them to take-part in the study. Interviews with both HCPs and patients will seek to understand the burden associated with any outcomes measures.

***Intervention acceptability.*** Acceptability is the perception among stakeholders that a given treatment, service, practice, or innovation is agreeable, palatable, or satisfactory ^71^. Acceptability should be assessed based on the participants’ direct experience or knowledge of various dimensions of the intervention delivered.

Intervention acceptability will be measured quantitively with HCPs and patients using the Theoretical Framework of Acceptability-Based Questionnaire (TFA-BQ). The validated generic TFA-BQ was developed as an adaptable tool to measure acceptability across various healthcare interventions ^74^. Questions on the seven component constructs (affective attitude, burden, ethicality, intervention coherence, opportunity costs, perceived effectiveness and self-efficacy) are adapted for the KNEE-DEeP study and each rated using a five-point Likert scale ranging from ‘Strongly disagree’ to ‘Strongly agree’.

Qualitative data collection with HCPs will explore the acceptability of the HCP intervention and their experience of the training workshops. How acceptable the HCPs perceived the patient intervention to be will also be discussed during these interviews. Patient interviews to explore acceptability will focus on understanding if patients liked and valued the various components of the KNEE-DEeP intervention as it was delivered.

***KNEE-DEeP fidelity.*** Fidelity is the degree to which an intervention was implemented as the developers of the intervention intended ^71, 75^. Fidelity of delivery and receipt of the KNEE-DEeP intervention will be assessed using quantitative and qualitative data collection. While it is not practical to measure directly GPs delivery of the components of the ‘enhanced consultation’, an indirect or proxy measure will be used in the form of two checklists. GPs will indicate their delivery of the four components of the ‘enhanced consultation’ on a checklist as part of completing the ‘best practice’ physiotherapy referral form. Patient participants will complete a checklist as part of their baseline questionnaire, indicating receipt of these intervention components during the GP consultation. At the end of each session, physiotherapists delivering the KNEE-DEeP ‘best practice’ session will complete treatment logs. This consists of a checklist documenting the key session components (e.g., clinical assessment, exploring beliefs, aspects of patient education, self-management skills, goal setting etc).

Qualitative data on aspects of fidelity, including delivery and receipt of HCP training, delivery of the patient intervention by HCPs and enactment of the clinical intervention by patients, will be gathered during interviews with a subset of patients and HCPs.

***Preliminary efficacy.*** Preliminary efficacy will be assessed by the change in the primary clinical outcome measure of self-reported knee-function. All primary and secondary clinical outcome measures are described below.

**Baseline descriptive data**

Demographic and descriptive data is collected using a baseline questionnaire. Table 3 includes a summary of descriptive data from patient participants. Descriptive data from GP participants will include: i) age; ii) gender; iii) years qualified as a GP; iv) practice size and location; v) special clinical interests, and; vi) any additional musculoskeletal training. Descriptive data from physiotherapist participants will include: i) age; ii) gender; iii) years qualified as a physiotherapist; iv) grade and v) special clinical interests.

**Patient clinical outcomes**

Selected clinical outcome measures are recommended by Outcome Measures in Rheumatology (OMERACT), Osteoarthritis Research Society International (OARSI). These include pain, physical function, quality of life, global assessment of the target joint, and adverse events ^76^. Table 3 provides the full list of clinical outcome measures and time points of assessment.

***Knee function.*** Self-reported knee function will be assessed using the Knee Injury and Osteoarthritis Outcome Score Physical Function Short Form (KOOS-PS) ^77^. This 7-item measure of physical function is derived from the activities of daily living and sport/recreation subscales of the Knee Injury and Osteoarthritis Outcome Score (KOOS). The KOOS-PS has been shown to be valid and reliable for use in people with knee OA. Scores range from 0 to 100 (extreme problems to no problems) ^77^.

***Knee pain.*** Knee pain will be measured by the nine item pain subscale of the KOOS. Questions on this subscale pertain to the previous 7 days. It is scored (0 to 100) and reported individually ^78^. This KOOS subscale is a valid, responsive and reliable measure that have been used extensively in knee OA and knee arthroscopy trials ^79^.

***Pain self-efficacy.*** The 2-items on the shortened form of the Pain Self-Efficacy Scale Questionnaire (PSEQ-2) reflect confidence in one's ability to work and lead a normal life despite pain ^80^. Items are scored on a 7-point Likert scale from "Not at all confident" to "Completely confident", with scores ranging from 0 (no confidence) to 12 (maximal confidence to perform activity despite pain). PSEQ-2 is valid and reliable and considered responsive enough to be used in a research context to evaluate changes after an intervention for chronic musculoskeletal disorders ^81^.

***Fear-avoidance beliefs.*** A modified version of the four-item fear avoidance beliefs about physical activity subscale (FABQ-PA) will be used ^82^. Participants will rate their beliefs about the activity from 0 (completely disagree) to 6 (completely agree) and scores range from 0 to 24. While not formally validated in the knee, other studies have used this modified version in people with knee pain ^83, 84^. The word “back” has been substituted with the word “knee,” and the physical activities examples have been substituted with “running, walking, kneeling, and driving.”

***Health related quality of life.*** The EuroQol Five-Dimensional Questionnaire (EQ-5D-5L) measures across five dimensions (mobility, self-care, usual activities, pain/ discomfort and anxiety/depression). The response options range from no problems (1) to unable or extreme problems (5). Overall health status is rated on a visual analogue scale from 0 (worst imaginable health) to 100 (best imaginable health) ^85^. Validity and reliability has been demonstrated in people with knee osteoarthritis populations ^86^.

***Global rating of change.*** Self-rated overall level of improvement in knee symptoms (pain and physical function) will be assessed. Participants will be asked to respond to the questions: ‘Overall, how has your knee pain changed since the start of the study?’ and ‘Overall, how has your knee function changed since the start of the study?’ using Likert scale (7-point) ranging from ‘much worse’ to ‘much better’.

***Satisfaction with care.*** Participants will be asked to rate the “overall satisfaction with the care you received in this study” using a 7-point scale ranging from “extremely unsatisfied” to “extremely satisfied”

***Adherence to the exercise and physical activity plan.*** Adherence to the exercise and physical activity plan agreed with the physiotherapists will be rated by the patient participant on an 11-point Numerical Rating Scale (0 = not at all and 10 = completely as instructed).

***Recovery expectations.*** Expectation of recovery are assessed using a 7-point ordinal scale in response to the question “How much do you expect your knee problem to change in the next 6 months?” ranging from ‘completely recovered’ to ‘worse than ever’.

***Use of health care and co-interventions for knee pain.*** A self-report checklist will record visits to health care providers (additional physiotherapy and GP consultations, other health professional or secondary care consultations), injection use, complementary therapies and imaging between baseline to 12 weeks and at 6-month follow-up using a customised questionnaire.

***Change in pain medication usage.*** Self-report change in pain medication use from baseline to 12-week and 24 follow-up will be assessed with a 7-point Likert scale (much less to much more).

***Number of working days missed.*** The number of work days (paid employment or unpaid work) missed due to the knee problem since baseline will be recorded.

***Adverse events.*** Defined as any problem that participants believe was caused by the intervention (exercise or advice) and requiring them to seek treatment and/or lasted for two or more days. Adverse events reported to the research team by participating patients, GPs or physiotherapists will be recorded in an adverse events log. Follow-up questionnaire at 12 weeks and 6 months will specifically ask patients about adverse events by including an open probing question. All qualitative interviews conducted with participants will ask about adverse events.

### Data collection and analysis plan

**Data analysis plan**

As this is a feasibility study, the main focus will be on feasibility and implementation outcomes to inform decisions about further evaluation and a fully-powered trial. For the process evaluation, insights into intervention feasibility, acceptability and the feasibility of study procedures will be identified from coded interview transcripts and survey results will be analysed using descriptive statistics. These findings will be integrated by accompanying quantitative data with qualitative findings in a joint display ^87^. A more detailed description of quantitative and qualitative analysis is included below.

**Quantitative data collection and analysis**

Data relating to study procedures (recruitment, retention etc) will be collected throughout. Participants will be invited to complete the baseline and follow-up questionnaires via an online link to Microsoft (MS) Forms. Data from MS Forms will be stored electronically on UL’s secure OneDrive cloud storage, exported to Microsoft Excel. Participants will also have the option of completing paper questionnaires, if this is their preference. Data from paper based questionnaires will be transferred by a researcher to Microsoft Excel. Demographic data from GPs and physiotherapists will be collected following study enrolment at the training workshops by paper questionnaire.

Follow-up questionnaires will be sent to patient participant at 12 weeks and 6 months. For those who do not respond, at least one reminder will be sent, via email or by post. For those who do not respond to the reminder, a telephone follow-up will be used to contact participants. Participating GPs and physiotherapists will complete a follow-up questionnaire at the conclusion of recruitment and intervention delivery.

Participants’ characteristics and implementation outcomes will be described using descriptive statistics such as means, standard deviations and ranges for quantitative variables, and counts and proportions for categorical variables. Descriptive statistics will also be used to measure change in outcome measures from baseline to 12 weeks and 6-month time-points and results will be treated as exploratory. Means and confidence intervals of the KOOS-PS will be calculated in order to inform the sample size calculation for a future definitive RCT.

**Qualitative data collection and data analysis**

***Patient interviews.*** Semi-structured interviews will be conducted by a female researcher with a background in qualitative methods, and who is not directly involved in the study. Interviews will be by telephone or online, depending on participant preference. Ten to 12 of the patient participants will be purposively recruited with the aim of interviewing a diverse sample, based on clinical characteristics, and participants from a range of participating GP practices. Interviews will be conducted within a month of receipt of the ‘best practice’ physiotherapy intervention, where possible, and last for approximately thirty minutes. The interview topic guide will focus on acceptability and feasibility of the study intervention and procedures, as outlined above, and also include : 1) patients’ experience of receiving the intervention from the GP and the physiotherapist; 2) perception of its benefits, 3) barriers to engagement with aspects of the intervention; 4) suggested improvements on aspects of the intervention or study design.

***HCP interviews.*** Qualitative data will be collected from six to eight GPs on conclusion of the patient recruitment phase. Data collection will be via a combination of one to one interviews and a small focus group moderated by clinician researcher not directly involved with the study. The dynamics of the focus group discussion should help promote debate and a range of views about the KNEE-DEeP study intervention ^88^, yielding rich data on acceptability and feasibility. Interviews will take place either in-person or online, depending on participant preference *.* Five physiotherapists who delivered the physiotherapy intervention will also be interviewed. The topic guides will explore the experiences of those HCPs participating in the study and issues of implementation relating to acceptability, feasibility, fidelity, as outlined in the outcome section above.

Transcription will be undertaken in parallel to data collection. Transcripts will be de-identified and offered to participants for approval or amendment. The NVivo qualitative analysis software programme ^89^ will be used to support data analysis and enable an audit trail of analysis decisions. Open coding of the data set will be undertaken before using thematic analysis ^90^. The research team will code the data, with peer coding of select transcripts, to enable cross-coding comparisons, before identifying themes and sub-themes. The wider research team including the PPI group, will contribute to interpretation the findings.

**Data management plan and study oversight**

Each participant will be assigned a unique study identifier. All electronic data collected via online questionnaires or manually entered into Microsoft Excel will be transferred automatically and securely stored on University of Limerick’s cloud storage (which is GDPR compliant and uses a two-step sign-in procedure to ensure security) in pseudo-anonymised format. A quality check of 20% of the data will be completed by an independent researcher. The PI will maintain the key to this pseudo-anonymisation and this will be stored securely in UHK. Only the study team will have access to pseudo-anonymised data. Written data containing participant information will be kept to a minimum and any information will be scanned and transferred to electronic format and stored securely with other study information. Written data pertaining to the physiotherapists ‘best practice’ session will be retained in UHK and filed with the patient’s clinical notes. Information from checklist documenting the key elements of the session will be pseudo-anonymised and transferred to electronic format. Consent forms will be stored separately to other data for six months after study completion. Data will then be anonymised and retained for another 7 years. Deidentified data will be made available on reasonable request to the PI after publication according to the University of Limerick Research Data Management Policy.

The Trial Management Committee (TMC) will oversee the day to day running of the trial and have responsibility for data monitoring. This committee will comprise of the PI and five grant collaborators. An independent Trial Steering Committee (TSC) will oversee the study. This includes an independent chairperson, two other independent members and a lay representative. The PI (HOL), and another member (KMC) of the TMC will attend the TSC meetings and report progress. The TSC meetings will be scheduled approximately every three months, and it is anticipated the committee will meet five times over the course of the study. Safety considerations associated with this trial are minimal because education and exercise for knee pain are associated with minimal adverse events. Any serious adverse events will be recorded and reported to both the ethics committee and the TSC. The committee will have responsibility for making decisions about modifying or discontinuing the intervention in response to any reported or potential harms. As this is a low risk trial, the Steering Committee will also act as the Independent Data Monitoring Committee (IDMC).

**Progression criteria**

Progression criteria were developed for this study to aid subsequent decisions around the need for further developmental work or proceeding to a full RCT. The criteria which focus on key feasibility and acceptability variables and are presented in the extended data (File J, https://osf.io/fwy36/). Development of these benchmarks were guided by existing literature ^91^, and the research team’s experience and knowledge of the local context.

### Dissemination plan

Study outcomes will be widely disseminated through a variety of sources. Feasibility study results will be presented at relevant conferences and published in a relevant peer-reviewed open-access journal. Patient participants will be provided with a lay summary of findings. Other wider dissemination strategies will be explored with the PPI group. Findings will also be disseminated through GP and physiotherapy professional networks in Ireland.

## Discussion

It is well established that non-surgical treatment is the optimal approach for a degenerative meniscal tear or early degenerative knee pain. Despite this, patients with this diagnosis continue to be referred to secondary care for a surgical opinion, with inadequate referral or access to first line treatment such as physiotherapy or exercise therapy. This study will seek to address some of the barriers that have contributed to the gap between the evidence and the care currently delivered in routine clinical practice in the Irish healthcare system.

In preparation for this study, extensive qualitative research and stakeholder consultation has been undertaken to develop the intervention that will be tested. This study will establish whether our intervention is feasible to deliver in a real world clinical setting and acceptable to health professionals and patients. It will provide insights into treatment fidelity and the feasibility of study procedures such as patient and HCP recruitment and retention. Study findings will help inform the development of a future large-scale research trial or form the basis for further developmental work.

This cohort of patients with early degenerative knee pain, without evidence of established OA, are an understudied population in clinical studies. Previous studies delivering conservative treatment to people with degenerative knee disease frequently limit their participation to people with established OA, e.g. with a three-month history of almost daily knee pain ^92, 93^. In those with early degenerative changes or meniscal pathology the focus to date has been on investigating arthroscopic meniscectomy. While the strong recommendations against surgery for degenerative meniscal pathology are gradually translating to practice, clinicians can feel at a loss as to how to best deliver non-surgical management ^30^. Multiple barriers exist, including at both the patient and service levels. This study seeks to address this gap in a unique way by developing a bespoke intervention for this cohort of patients in addition to improving the skills and confidence of HCPs delivering this care. The KNEE-DEeP intervention also represents a novel approach by trialling the delivery of a single session approach. There is mounting evidence supporting the use of a single session for shoulder and spinal pain ^45, 46^, but this has not been investigated to date in the knee and it’s acceptability to HCPs and patients is unknown. Identifying a sub-population of people with knee pain who might benefit from this approach could have far reaching consequences for the use of resources in the management of musculoskeletal pain.

### Trial status

This is version 1 of the protocol (December 2024). Recruitment of patient participants commenced in June 1 ^st^ 2024 and is ongoing. The study is due for completion in June 2025. Deviations from this protocol will be submitted to the ethics board, updated on the trial registry ( clinicaltrials.gov), and described in the final manuscript of the results.

### Name and contact of the trial sponsor

University of Limerick, Castletroy Co. Limerick, Ireland.

## Abbreviations

**BCW:** Behavioural Change Wheel

**DMT:** degenerative meniscal tear

**GP:** General Practitioners

**HCP:** Health Care Professionals

**HRB** Health Research Board

**HSE:** Health Services Executive

**KNEE-DEeP:** Knowledge Translation and Exercise for Degenerative Meniscal Pathology and Early Osteoarthritis

**MRC:** Medical Research Council

**NICE:** National Institute for Health and Care Excellence

**OA:** Osteoarthritis

**PI:** Principal Investigator

**RCT:** Randomised Controlled Trial

**TDF:** Theoretical Domains Framework

**TMC:** Trial Management Committee

**TSC:** Trial Steering Committee

## Ethics and informed consent

Ethical approval for this study was granted on the 5 ^th^ December 2023 by Clinical Research Ethics Committee of the Cork Teaching Hospitals ( *Reference Number: ECM 4 (h) 24/10/2023 & ECM 3 (d) 05/12/2023)*. This committee has remit to provide approval for studies conducted within the HSE Community Health Organisation for Cork/Kerry. This study involves human participants and will adhere to the Declaration of Helsinki ^56^. Prior to study recruitment all participants will be provide written informed consent, in line with the Data Protection Act 2018 ^57^. Eligible participants will be invited to ask questions about the study and a time period of several days will elapse between receiving information about the study and making their final decision about whether to participate.

## Data Availability

No data are associated with this article. Open Science Framework: Knowledge Translation and Exercise for Degenerative Meniscal Pathology and Early Osteoarthritis (KNEE-DEeP): A Single Arm Feasibility Study. https://doi.org/10.17605/OSF.IO/FWY36 ^58^. This project contains the following extended data: File A. KNEE-DEeP Checklist CONSORT-extension-Pilot-and-Feasibility File B. KNEE-DEeP Checklist SPIRIT File C. KNEE-DEeP_GP_Info_Leaflet and Consent Form.docx File D. KNEE_DEeP_Physio_Info_Leaflet and Consent Form.docx File E. KNEE_DEeP_Patient_Info Leaflet and Consent.docx File F. Patient Advice Leaflet_Understanding_my_knee_pain.pdf File G. KNEE-DEEP Physio Referral Form.docx File H. Key Educational Messages delivered by GP.pdf File I. KNEE-DEeP Exercise Ladder.pdf File J. KNEE-DEeP Progression Criteria.docx Data are available under the terms of the Creative Commons Zero "No rights reserved" data waiver (CC0 1.0 Universal). Open Science Framework: SPIRIT checklist. “Knowledge Translation and Exercise for Degenerative Meniscal Pathology and Early Osteoarthritis (KNEE-DEeP): A Single Arm Feasibility Study”. https://doi.org/10.17605/OSF.IO/FWY36 ^58^ Data are available under the terms of the Creative Commons Zero "No rights reserved" data waiver (CC0 1.0 Universal).
