# Supplementary material for: Knowledge translation and exercise for degenerative meniscal pathology and early osteoarthritis  (KNEE-DEeP): Protocol for a single arm feasibility study
Source: HRB Open Res. 2025 Jan 24;8:14. [Version 1] doi: 10.12688/hrbopenres.14049.1 (PMC11868751; doi:10.12688/hrbopenres.14049.1)
Supplement: Peer review history

# Reviewer report: Reviewer response for version 1

**DOI:** 10.21956/hrbopenres.15426.r45076

**Published:** 2025-02-27

**Authors:** Alison J. Gibbs

**Affiliations:** 1 La Trobe University, Bundoora, Australia

This is single arm feasibility study exploring the feasibility and acceptability of an enhanced GP consultation, patient resources and physiotherapy consultation to improve evidence-based care for degenerative meniscal pathology and early osteoarthritis. A very detailed and thorough, well-presented protocol.

 A few minor points: 

 -The methods state if unsure will refer for x-ray to determine if moderate to severe OA, what is the criteria for deciding when to x-ray to exclude moderate-severe radiographic -OA, as pain is not necessarily correlated with radiographic severity?

 -A checklist for physiotherapists relating to excluding moderate-severe OA is mentioned-could this be provided?

 -For the qualitative analysis and coding-what is the underlying philosophical approach, are the research team coding independently?

 -Table 3 timelines for outcomes-no timeline provided for when symptom duration and working days missed will be collected

Is the study design appropriate for the research question?

Yes

Is the rationale for, and objectives of, the study clearly described?

Yes

Are sufficient details of the methods provided to allow replication by others?

Yes

Are the datasets clearly presented in a useable and accessible format?

Yes

Reviewer Expertise:

Osteoarthritis

I confirm that I have read this submission and believe that I have an appropriate level of expertise to confirm that it is of an acceptable scientific standard.

# Reviewer report: Reviewer response for version 1

**DOI:** 10.21956/hrbopenres.15426.r45074

**Published:** 2025-02-24

**Authors:** Jeanette M Thom

**Affiliations:** 1 University of Sydney, Sydney, Australia

The authors have prepared an excellent manuscript that is clear and detailed.

 Minor points:

 It is not clear if you would like the GPs to be recruited from a range of different locations or would prefer them to mostly come from a few clinics with multiple GPs. Also, it is not clear how the physios are then linked to the same patients.

 Does providing patients with the information that the service will be ‘best practice’ influence their thoughts on the outcomes? If so, how are you going to manage this?

 Are there other co-morbidities that you would exclude people from the study, e.g. heart disease, T2D?

 Clarity of who and how gaining informed consent from patients without being coerced from their GP. Do they receive their ‘GP best practice consultation’ prior to enrolling in the study?

 Table 3 – some outcome measures have no information regarding when they will be collected (e.g. number of working days missed)

 Page 15: Why not invite all patient participants and GPs to be recruited for the interviews? What if your sub-sampling does not reach data saturation? Are you not interviewing the physiotherapists?

Is the study design appropriate for the research question?

Yes

Is the rationale for, and objectives of, the study clearly described?

Yes

Are sufficient details of the methods provided to allow replication by others?

Yes

Are the datasets clearly presented in a useable and accessible format?

Yes

Reviewer Expertise:

Exercise Physiology and musculoskeletal conditions

I confirm that I have read this submission and believe that I have an appropriate level of expertise to confirm that it is of an acceptable scientific standard.
